# Necrolytic migratory erythema as the first manifestation of pancreatic neuroendocrine tumor

**DOI:** 10.1186/1477-7819-12-220

**Published:** 2014-07-17

**Authors:** Sheng-li Wu, Ji-gang Bai, Jun Xu, Qing-yong Ma, Zheng Wu

**Affiliations:** 1 Department of Hepatobiliary Surgery, the First Affiliated Hospital of Xi’an Jiaotong University School of Medicine, No. 277, Yanta West Road, 710061 Xi’an, Shaanxi, P.R. China

**Keywords:** Necrolytic migratory erythema, Glucagonoma, Pancreatic neuroendocrine tumors

## Abstract

Necrolytic migratory erythma (NME) is an obligatory paraneoplastic syndrome. Here we describe a woman admitted to the dermatology ward with NME which was later found to be associated with glucagonoma, a slow-growing, rare pancreatic neuroendocrine tumor. Even more rarely, the tumor was located in the pancreas head, while most of such lesions are located in the distal pancreas. The diagnosis of this rare tumor requires an elevated serum glucagon level and imaging confirming a pancreatic tumor. After surgical removal of the tumor, the patient’s cutaneous and systemic features resolved. It is therefore imperative that clinicians recognize NME early in order to make an accurate diagnosis and to provide treatment for this rare tumor.

## Background

Necrolytic migratory erythema (NME) is a rare skin disorder which was first described by Becker et al. in 1942 [1]. Erythematous scaly lesions with centrifugal growth characterize the clinical appearance of the disease. Perineum, distal extremities, lower abdomen, and face are the most commonly affected sites [2]. NME is an obligatory paraneoplastic syndrome. Most of NME cases are associated with pancreatic neuroendocrine tumors (PNETs). PNETs are rare neoplasms representing <5% of all pancreatic malignancies with an estimated incidence of 1 to 1.5 cases/100,000 [3] and are classified by the hormonal products that they produce. Glucagonomas, considered among the rarest of PNETs, produce a well defined clinical syndrome characterized by NME, diabetes mellitus, glossitis, anemia, and weight loss [4]. In this paper, we describe a woman admitted to the dermatology ward with NME, which was later found to be associated with glucagonoma located at the head of the pancreas. We will describe NME, the glucagonoma syndrome and how to recognize and treat this rare tumor.

## Case presentation

**Figure 1:**
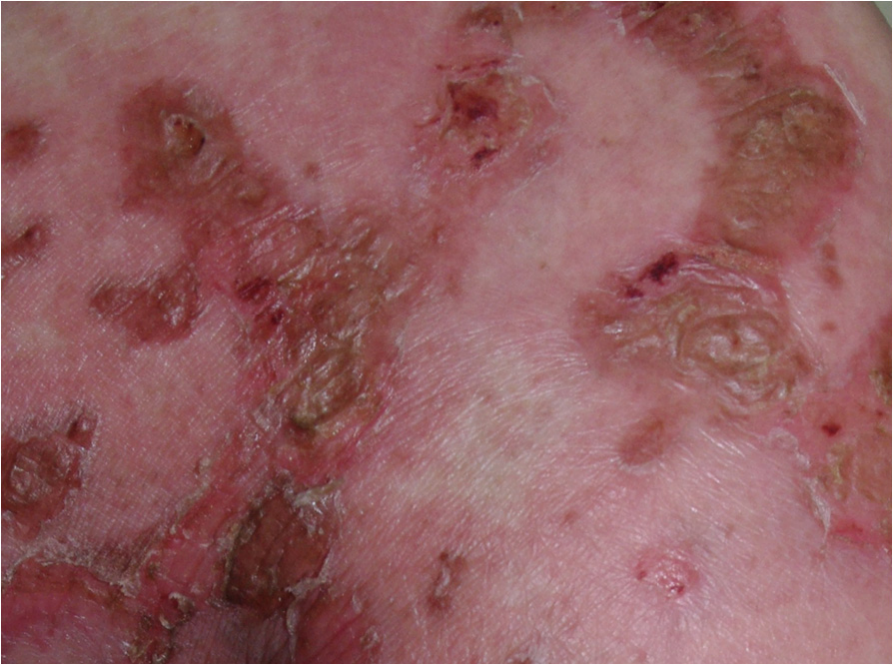
Close-up view of well demarcated erythematous plaques, with fragile vesicles on gluteal area.

A 44-year-old woman presented with a 3-month history of pruritic rash of the extremities and oral cavity. The rash progressed to involve the groin, abdomen, axillae, and gluteal region. She was found to have well demarcated, erythematous plaques in these regions. Tiny fragile vesicles were observed at the margins of several plaques (Figure 1). At this time, the patient was admitted to the dermatology ward for further evaluation. The patient underwent allergen patch testing, which was negative, and was treated with topical steroids without clinical improvement. Skin biopsy revealed psoriasiform acanthosis and abrupt necrosis of the upper layers of stratum; whereas the lower half of epidermis appears viable, the detached necrolytic portion appears pale with pyknotic nuclei. Perivascular lymphocytic infiltration and scattered extravasated red blood cells were present in the upper dermis.

**Figure 2:**
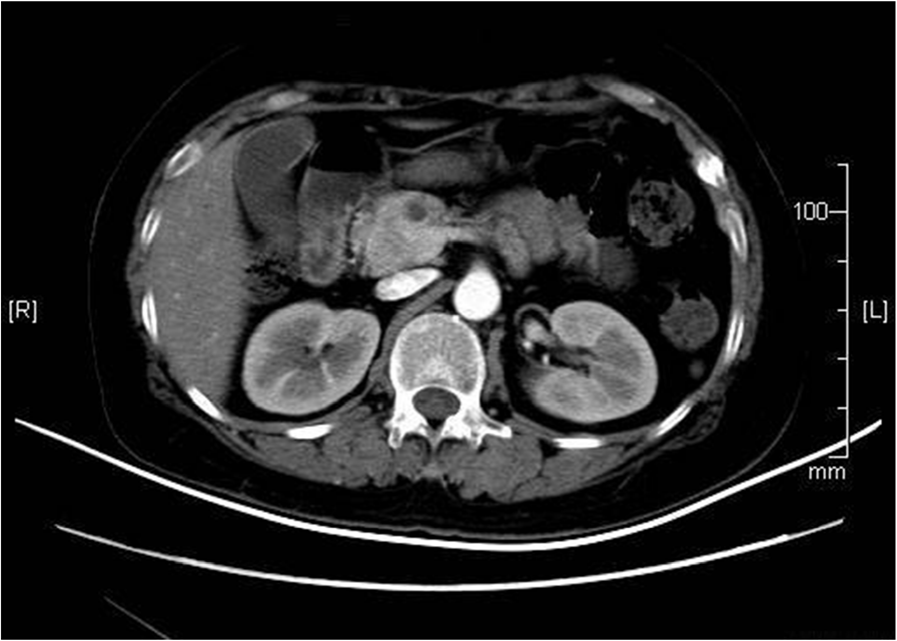
**Contrast-enhanced computed tomography scan of the abdomen.** A 3-cm nodular mass in the head of the pancreas was seen. There was no evidence of metastases.

Based on these findings, NME was considered. A glucagon level was obtained and a contrast-enhanced CT scan was conducted. Her level of serum glucagon was elevated at 720 ng/L (normal values 40 to 130 ng/L) (Table 1) and the scan revealed a 3-cm enhancing pancreatic head mass (Figure 2). Thus, a referral to the hepatobiliary department was made.

**Table 1:** Laboratory values on the day of hospital admission and hospital day 14 (post-operative day 7)

**Serum laboratory studies**	**Admission values**	**Post-operative values**	**Normal values**
Glucose (mg/dL)	132	85	60–99
Glucagon (ng/L)	720	233	40–130
Chromogranin A (ng/mL)	243	87	<96
Glycosylated hemoglobin (%)	6.3	4.2	<5.7

On admission, she had stable vital signs. Admission laboratory measurements demonstrated hyperglycemia (132 mg/dL, normal values 60 to 99 mg/dL) and anemia (hemoglobin 11.4, normal values 12 to 16 g/dL). Serum levels of chromogranin A, a tumor marker for neuroendocrine tumors, were elevated (243 ng/mL, normal value <96 ng/mL), as were levels of glycosylated hemoglobin (6.3%, normal value <5.7%) and the patient was diagnosed with diabetes mellitus.

On hospital day 7, she underwent pancreaticoduodenectomy. Pathology confirmed a pancreatic neuroendocrine carcinoma with evidence of vascular and perineural invasion, consistent with a diagnosis of a glucagonoma. In the days following her operation, the patient was admitted to the ICU, where she faced pancreatic fistula that resolved with non-operative care. Her glucagon and glucose blood levels dropped dramatically post-operatively (Table 1). One week after the surgery, the patient's cutaneous lesions almost disappeared. Currently, more than 1 year post-operatively, there was no sign of cutaneous or systemic disease.

## Conclusions

Glucagonoma is a slow growing PNET, which is often heralded by paraneoplastic phenomena. The key features of the glucagonoma syndrome, a rare disorder with an estimated incidence of 1 in 20 million [5], are NME and diabetes mellitus [6]. Other common manifestations include weight loss, stomatitis, steatorrhea, diarrhea, thromboembolic tendency, anemia, and neuropsychiatric features [7]. Males and females are equally affected, with an age peak presentation in the fifth decade of life [8]. The diagnosis of a glucagonoma requires an elevated serum glucagon level and imaging confirming a pancreatic tumor. Unfortunately, delays in diagnosis are common for this slow growing tumor. It may proceed at least 1 year before a correct diagnosis is made [9]. At least half of patients will have metastatic disease when diagnosed [10]. Delays in diagnosis have been attributed to the extreme rarity of the tumor, under-recognition of the dermatologic presentation, the difficulty making the diagnosis of NME on biopsy, and the lack of specificity of the other symptoms [11].

NME is the most specific feature of the syndrome, and is the presenting symptom in approximately 70% of patients [9]. The exact cause of NME remains unknown. Although normalization of glucagon level by resection of the tumor usually results in a rapid disappearance of the skin disorder [12], elevation of serum glucagon levels alone cannot explain all of the skin findings, since hyperglucagonemia occurring in other situations, such as trauma, burns, diabetic ketoacidosis starvation and cirrhosis, does not lead to a typical rash [13]. Hypoaminoacidemia or other nutritional deficiencies, such as essential fatty acids or zinc, might also contribute to the occurrence of NME because of the cutaneous histologic similarities between these deficiency states and this syndrome [14,15]. The early recognition and correct diagnosis of NME is important because it can be the only and the first manifestation of the glucagonoma syndrome. However, the correct diagnosis of NME is challenging as the lesions of the NME may be often misinterpreted as contact dermatitis, intertrigo, inverse psoriasis, zinc deficiency, and other nutritional deficiencies [7].

Diabetes mellitus is another common finding. Glucagon has many important effects on glucose metabolism. It stimulates hepatic gluconeogenesis, inhibits glycolysis and glycogen synthesis. Case studies suggest that 80% of patients with glucagonomas will eventually develop the disease [12].

Glucagonomas are typically distal pancreatic tumors, generally large in size due to late detection, often measuring between 4 and 10 cm [16]. However, the tumor in our patient was located in the head of the pancreas, which is uncommon for this disease. Moreover, the tumor was detected in its early stage due to the prompt and accurate diagnosis.

Contrast-enhanced CT is recommended to identify the number and location of tumors. Somatostatin receptor scintigraphy may be obtained if there is concern for distant disease [17]. The best and the most effective treatment strategy should aim to lower serum glucagon levels. A complete resection of the tumor is the best option because of its weak response to chemotherapy [14]. Patients who underwent resection had significantly longer median survival than patients who did not receive surgery, even when diagnosed with later stages of disease [18].

## Consent

Written informed consent was obtained from the patient for the publication of this report and any accompanying images.

## Abbreviations

CT: computed tomography; NME: necrolytic migratory erythma; PNET: pancreatic neuroendocrine tumor.

## Competing interests

The authors declare that they have no competing interests.

## Authors’ contributions

SLW and ZW conceived and designed the study. JGB and JX collected the clinical data. SLW and QYM wrote the manuscript. All authors approved the final manuscript.

## References

[B1] Becker SW Brennan BB Benign and malignant cutaneous tumors in the elderly Arch Dermatol 1961 83 262 271 14448530 10.1001/archderm.1961.01580080092010

[B2] Afsharfard A Atqiaee K Lotfollahzadeh S Alborzi M Derakhshanfar A Necrolytic migratory erythema as the first manifestation of glucagonoma Case Rep Surg 2012 2012 974210 22970401 10.1155/2012/974210 PMC3434377

[B3] Batcher E Madaj P Gianoukakis AG Pancreatic neuroendocrine tumors Endocr Res 2011 36 35 43 21226566 10.3109/07435800.2010.525085

[B4] Edney JA Hofmann S Thompson JS Kessinger A Glucagonoma syndrome is an underdiagnosed clinical entity Am J Surg 1990 160 625 628 2252125 10.1016/s0002-9610(05)80761-5

[B5] Wermers RA Fatourechi V Wynne AG Kvols LK Lloyd RV The glucagonoma syndrome. Clinical and pathologic features in 21 patients Medicine 1996 75 53 63 8606627 10.1097/00005792-199603000-00002

[B6] Johnson SM Smoller RB Lamps LW Horn TD Necrolytic migratory erythema as the only presenting sign of a glucagonoma J Am Acad Dermatol 2003 49 325 328 12894090 10.1067/s0190-9622(02)61774-8

[B7] Lobo I Carvalho A Amaral C Machado S Carvalho R Glucagonoma syndrome and necrolytic migratory erythema Int J Dermatol 2010 49 24 29 20465606 10.1111/j.1365-4632.2009.04220.x

[B8] Doherty GM Rare endocrine tumours of the GI tract Best Pract Res Clin Gastroenterol 2005 19 807 818 16253902 10.1016/j.bpg.2005.05.004

[B9] Eldor R Glaser B Fraenkel M Doviner V Salmon A Gross DJ Glucagonoma and the glucagonoma syndrome - cumulative experience with an elusive endocrine tumour Clin Endocrinol 2011 74 593 598 10.1111/j.1365-2265.2011.03967.x 21470282

[B10] Echenique-Elizondo M Tuneu Valls A Elorza Orúe JL Martinez De Lizarduy I Ibáñez Aguirre J Glucagonoma and pseudoglucagonoma syndrome JOP 2004 5 179 185 15254346

[B11] Halvorson SA Gilbert E Hopkins RS Liu H Lopez C Chu M Martin M Sheppard B Putting the pieces together: necrolytic migratory erythema and the glucagonoma syndrome J Gen Intern Med 2013 28 1525 1529 23681843 10.1007/s11606-013-2490-5 PMC3797362

[B12] Van Beek AP De Haas ER Van Vloten WA Lips CJ Roijers JF Canninga-van Dijk MR The glucagonoma syndrome and necrolytic migratory erythema: a clinical review Eur J Endocrinol 2004 151 531 537 15538929 10.1530/eje.0.1510531

[B13] Adam DN Cohen PD Ghazarian D Necrolytic migratory erythema: case report and clinical review J Cutan Med Surg 2003 7 333 338 14738101 10.1007/s10227-002-0127-0

[B14] Alexander EK Robinson M Staniec M Dluhy RG Peripheral amino acid and fatty acid infusion for the treatment of necrolytic migratory erythema in the glucagonoma syndrome Clin Endocrinol 2002 57 827 831 10.1046/j.1365-2265.2002.01660.x 12460334

[B15] Chastain MA The glucagonoma syndrome: a review of its features and discussion of new perspectives Am J Med Sci 2001 321 306 320 11370794 10.1097/00000441-200105000-00003

[B16] Akerström G Hellman P Surgical aspects of neuroendocrine tumours Eur J Cancer 2009 45 237 250 19775622 10.1016/S0959-8049(09)70039-5

[B17] Sundin A Garske U Orlefors H Nuclear imaging of neuroendocrine tumours Best Pract Res Clin Endocrinol Metab 2007 21 69 85 17382266 10.1016/j.beem.2006.12.003

[B18] Hill JS McPhee JT McDade TP Zhou Z Sullivan ME Whalen GF Tseng JF Pancreatic neuroendocrine tumors: the impact of surgical resection on survival Cancer 2009 115 741 751 19130464 10.1002/cncr.24065

